# Mycobacterial MazG Safeguards Genetic Stability *via* Housecleaning of 5-OH-dCTP

**DOI:** 10.1371/journal.ppat.1003814

**Published:** 2013-12-05

**Authors:** Liang-Dong Lyu, Bi-Kui Tang, Xiao-Yong Fan, Hui Ma, Guo-Ping Zhao

**Affiliations:** 1 CAS-Key Laboratory of Synthetic Biology, Institute of Plant Physiology and Ecology, Shanghai Institutes for Biological Sciences, Chinese Academy of Sciences, Shanghai, China; 2 Department of Microbiology and Li Ka Shing Institute of Health Sciences, The Chinese University of Hong Kong, Prince of Wales Hospital, Shatin, New Territories, Hong Kong SAR, China; 3 Anhui Key Laboratory of Infection and Immunity, Department of Life Science, Bengbu Medical College, Bengbu, China; 4 Shanghai Public Health Clinical Center Affiliated with Fudan University, Shanghai, China; 5 Key Laboratory of Medical Molecular Virology affiliated with the Ministry of Education and Health, Shanghai Medical College, Department of Microbiology, School of Life Sciences, Fudan University, Shanghai, China; 6 Shanghai-MOST Key Laboratory for Health and Disease Genomics, Chinese National Human Genome Center, Shanghai, China; National Institutes of Health, United States of America

## Abstract

Generation of reactive oxygen species and reactive nitrogen species in phagocytes is an important innate immune response mechanism to eliminate microbial pathogens. It is known that deoxynucleotides (dNTPs), the precursor nucleotides to DNA synthesis, are one group of the significant targets for these oxidants and incorporation of oxidized dNTPs into genomic DNA may cause mutations and even cell death. Here we show that the mycobacterial dNTP pyrophosphohydrolase MazG safeguards the bacilli genome by degrading 5-OH-dCTP, thereby, preventing it from incorporation into DNA. Deletion of the (d)NTP pyrophosphohydrolase-encoding *mazG* in mycobacteria leads to a mutator phenotype both under oxidative stress and in the stationary phase of growth, resulting in increased CG to TA mutations. Biochemical analyses demonstrate that mycobacterial MazG can efficiently hydrolyze 5-OH-dCTP, an oxidized nucleotide that induces CG to TA mutation upon incorporation by polymerase. Moreover, chemical genetic analyses show that direct incorporation of 5-OH-dCTP into *mazG*-null mutant strain of *Mycobacterium smegmatis* (*Msm*) leads to a dose-dependent mutagenesis phenotype, indicating that 5-OH-dCTP is a natural substrate of mycobacterial MazG. Furthermore, deletion of *mazG* in *Mycobacterium tuberculosis* (*Mtb*) leads to reduced survival in activated macrophages and in the spleen of infected mice. This study not only characterizes the mycobacterial MazG as a novel pyrimidine-specific housecleaning enzyme that prevents CG to TA mutation by degrading 5-OH-dCTP but also reveals a genome-safeguarding mechanism for survival of *Mtb in vivo*.

## Author Summary

The cellular nucleotide pool is a significant target for oxidation by reactive oxygen species and reactive nitrogen species. Misincorporation of these oxidized non-canonical nucleotides into DNA is known to cause mutations, and may be related to carcinogenesis, aging and neurodegeneration. Cells have evolved a group of bio-degradation housecleaning enzymes that may specifically eliminate certain non-canonical nucleotide from the nucleotide pool and thus prevent their incorporation into DNA. The most well-characterized housecleaning enzymes are the MutT-like proteins which specifically hydrolyze the oxidized purine nucleotides, such as 8-oxo-dGTP and 2-OH-dATP. Lack of MutT activity in cells leads to significant increase of AT-CG mutation and genetic instability. However, housecleaning enzymes specific for oxidized pyrimidine nucleotides are yet to be identified. Here we show that the dNTP pyrophosphohydrolase MazG from mycobacteria is a 5-OH-dCTP-specific housecleaning enzyme. Deletion of *mazG* in mycobacteria results in increased CG to TA mutation under oxidative stress and in the stationary phase of growth. Both biochemical and chemical genetic analyses demonstrate that 5-OH-dCTP is a natural substrate of mycobacterial MazG. Furthermore, deletion of *mazG* in *Mtb* leads to reduced survival in activated macrophages and in the spleen of infected mice. These results reveal a novel housecleaning pathway for mycobacteria to maintain genetic stability and survival *in vivo*.

## Introduction

Oxidative damage to DNA and the DNA precursors, deoxynucleotides (dNTPs) is an inevitable mutagenic challenge occurring in normal aerobic metabolism, generating a large amount of reactive oxygen species (ROS) as by-products during respiration or oxidation-reduction reaction [1]-[3]. Oxidative DNA damage is also an important innate immune response mechanism implemented by phagocytes, which produce large amount of ROS and reactive nitrogen species (RNS) as a bactericidal strategy to eliminate microbial pathogens [4], [5]. Increasing evidence shows that the nucleotide pool is a significant target for oxidative modification via ROS and substantial portion of the oxidative damage to genomic DNA is caused by incorporation of oxidized dNTPs from the nucleotide pool [3], [6], [7]. Due to their ambiguous conformation (*anti*/*syn*) compared to that of the canonical dNTPs, incorporation of oxidized dNTPs into DNA is known to cause mispairing and mutation, and may be related to carcinogenesis, aging and neurodegeneration [6], [8]-[10]. Recent studies also established that incorporation of oxidized dNTPs into DNA is a major causative mechanism for bacterial cell death induced by bactericidal antibiotics [11], [12]. Therefore, like the DNA repair enzymes, elimination of the oxidatively damaged dNTPs from the nucleotide pool is an important defense line for cells to maintain genetic stability.

Cells have evolved a group of non-canonical nucleotide-specific bio-degradation enzymes, named housecleaning enzyme, to eliminate the oxidized non-canonical dNTPs from the nucleotide pool and thus prevent their incorporation into DNA [13], [14]. These proteins belong to four structural superfamilies: 1) dUTPase, 2) ITPase, 3) Nudix (nucleoside diphosphate linked to an X moiety, or MutT-like) hydrolase, and 4) all-α NTP pyrophosphohydrolase (MazG NTP-PPase) [14]. The dUTPase and ITPase are NTP phosphatases that target dUTP, an intermediate during dTTP synthesis, and ITP/XTP, the deamination products of purine nucleotides, respectively. MutT is the best-studied Nudix hydrolase specific for oxidatively damaged nucleotides [14], [15]. *Escherichia coli* MutT is the first characterized Nudix enzyme with 8-oxo-dGTP and 8-oxo-GTP as its natural substrates. Deletion of *mutT* in *E. coli* results in increased AT to CG mutation in both DNA and mRNA [8], [16]. MTH1, the MutT-like protein in humans, is active against 8-oxo-dGTP, 8-oxo-dATP and 2-OH-dATP [17]. Depletion of *MTH1* in mice leads to a higher incidence of spontaneous tumorigenesis [18], while in human cells, MTH1 is involved in maintenance of genome stability and suppression of degenerative disorders such as neurodegeneration and carcinogenesis [6], [7], [19]. However, all the natural substrates for the MutT-like proteins that have been characterized in various organisms so far have been the oxidized purine nucleotides [15].

Oxidized pyrimidine nucleotides likely have a mutagenic effect similar to that of oxidized purine nucleotides. First, dCTP and dTTP can be oxidatively modified by ROS to form 5-OH-dCTP and 5-CHO-dUTP, respectively [20], [21]. Second, direct incorporation of 5-OH-dCTP or 5-CHO-dUTP into *E. coli* cells may cause an increase in mutation frequency, and both of these oxidized nucleotides may be mispaired with adenine rather than guanine leading to CG to TA mutation [10], [22]. Furthermore, 5-OH-dCTP is known to be incorporated into DNA more efficiently than 8-oxo-dGTP catalyzed by the exonuclease-free Klenow fragment [10]. Finally, it was found that the amount of 5-OH-dC in normal or oxidized cellular DNA is comparable to that of 8-oxo-dG [1], [23]. In addition to their role in mutagenesis, oxidized pyrimidine nucleotides also show a highly lethal effect on *E. coli*, indicating that these non-canonical nucleotides may disturb normal DNA replication and nucleotide metabolism [22]. Therefore, it is reasonable to conclude that cells have evolved housecleaning enzymes to eliminate oxidized pyrimidine nucleotides [10], [22]. However, although various enzymes responsible for the removal of oxidized pyrimidine in DNA molecules have been identified [24]–[26], the long-awaited housecleaning enzyme specific for elimination of oxidized pyrimidine nucleotides has yet to be characterized.

MazG-like proteins are widespread in all three domains of life and have been biochemically characterized as NTP-PPase while structurally categorized into the all-α NTP pyrophosphohydrolases superfamily unrelated to the MutT-like housecleaning enzymes [14], [27], [28]. It was found that *E. coli* MazG can regulate cellular (p)ppGpp levels and thus, may control programmed cell death under starvation conditions [29]. However, the mechanism whereby MazG regulates the cellular (p)ppGpp levels is still unclear. Structure-based modeling study of MazG from *sulfolobus solfataricus* suggested that 2-OH-dATP might be its most likely substrate and thus proposed, for the first time, a probable role of housecleaning for this enzyme [27]. Recently, it was reported that RS21-C6, a MazG-like enzyme in mice, showed a preference for degrading dCTP and its derivatives, with 5-I-dCTP as the most preferred substrate *in vitro* [30]. This attempt to identify a pyrimidine-specific housecleaning enzyme was echoed by structure-based analysis, which found that RS21-C6 binds to 5-methyl dCTP [31]. However, the natural substrate of the MazG family proteins remained unclear because none of the suggested functions involving regulation of pyrimidine synthesis [30], prevention of inappropriate DNA methylation [31], or antimutagenesis by degrading abnormal dCTP [27], [30] have ever been verified *in vivo*.

Although mycobacterial MazG has been characterized as a potent NTP pyrophosphohydrolase capable of hydrolyzing all canonical (d)NTPs *in vitro*, MazG can also hydrolyze dUTP and 8-oxo-dGTP, with their affinity to these substrates being similar to their affinity to the canonical dNTPs (*K_m_*∼1 mM). Therefore, it is unlikely that these non-canonical nucleotides are the natural substrate of the mycobacterial MazG *in vivo* [28]. In this study, we demonstrate that 5-OH-dCTP is a natural substrate of mycobacterial MazG by means of enzymatic and chemical genetic analyses. In addition to confirming the antimutator function of MazG, we show that deletion of *mazG* in the virulent *Mtb* strain H37Rv results in reduced survival in activated macrophage and mice. Our results reveal that mycobacterial MazG is a novel housecleaning enzyme involved in a pathway preventing the CG to TA mutation and ensuring the survival of *Mtb in vivo*.

## Results

### Mycobacterial *mazG* is an antimutator

**Figure 1:**
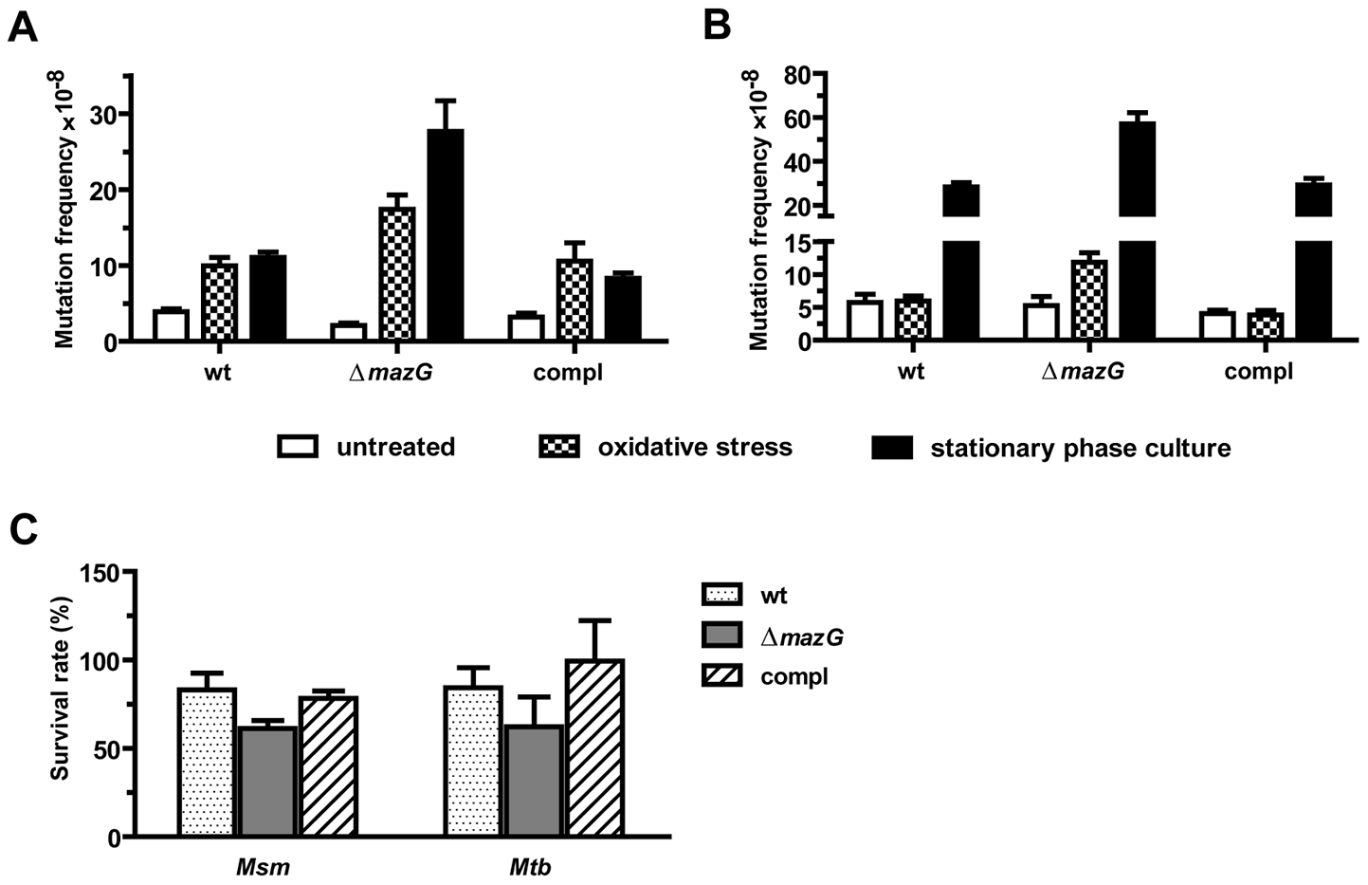
The antimutator role of MazG in *Msm* (A) and *Mtb* (B). Both the bacterial culture conditions and the methods for determination of mutation frequencies were illustrated in ***Materials and Methods*** in detail. The frequencies conferring resistance to rifampicin in wild-type (wt), *mazG*-null (Δ*mazG*) and the complemented mutant (compl) strains were determined in exponential phase (OD_600_∼0.5) with or without oxidative stress and in the stationary growth phase. Oxidative stress was induced by treating exponential phase cultures with 10 mM H_2_O_2_ for 5 h (*Msm*) or 24 h (*Mtb*). Stationary phase was at the 5^th^-day or 28^th^-day of culture for *Msm* or *Mtb*, respectively. (**C**) Survival rate of *Msm* and *Mtb* strains after exposure to H_2_O_2_. The numbers shown are mean ± S.E. of 3 independent experiments totaling 15 cultures of *Msm* and 6 of *Mtb*.

Previously, we demonstrated that lack of the MazG NTP-PPase activity in *Msm* strain mc^2^ 155 rendered the bacilli more susceptible to killing by hydrogen peroxide (H_2_O_2_) [28]. In order to test whether the oxidative stress resistant effect of the mycobacterial MazG is truly attributable to its potential housecleaning function in degrading certain oxidatively damaged dNTP(s), the spontaneous rifampicin-resistance mutation frequencies in wild-type and *mazG*-null (Δ*mazG*::*hyg*) *Msm* (bacterial strains used in this study are list in **Table S1**) were measured under different physiological conditions. We showed that the rifampicin-resistance mutation frequency in the *mazG*-null *Msm* increased 8.7-fold when treated with H_2_O_2_ (known to generate hydroxyl radicals which damage the dNTP pool [11], [32]), in contrast to merely 2.5 times increase in the wild-type *Msm* (**Figure 1A**). It was also found that, under the oxidative stress conditions, the expression level of *recA* and *dnaE2*, which is known to be elevated by damaged DNA (SOS response) [33], was 2-fold and 3-fold higher in the *mazG*-null *Msm* than that in the wild-type *Msm*, respectively (**Figure S1**). This suggests that, under oxidative stress, the *mazG*-null *Msm* suffers more genetic assaults than does the wild type. On the other hand, during the exponential phase of growth, the rifampicin-resistance mutation frequency of *mazG*-null *Msm* is comparable to that of the wild-type *Msm* (**Figure 1A**).

We also measured the rifampicin-resistance mutation frequency in the stationary phase of growth, a stage known to accumulate metabolic byproducts and mutations [34]. It was found that the rifampicin-resistance mutation frequency in 5-day-old *mazG*-null *Msm* cultures was 2.5 times greater than that in wild type (**Figure 1A**), suggesting a mutator phenotype of *mazG*-null *Msm* during the stationary phase of growth. A similar result was observed with 8-day-old cultures (data not shown), indicating prolonged incubation during the stationary phase does not further increase the mutation frequency in *mazG*-null *Msm*. To test whether *mazG* plays the same function in *Mtb*, we constructed a Δ*mazG*::*hyg* null mutation in the virulent *Mtb* strain H37Rv by means of allelic exchange and the deletion of this gene was confirmed by Southern blot (**Figure S2**). The *mazG*-null *Mtb* exhibited the same mutator phenotype as that of the *mazG*-null *Msm* (**Figure 1B**), showing a 2.5-fold increase in rifampicin-resistance mutation frequency compared to that of the wild type under oxidative stress or the stationary phase of growth.

In order to test the cytotoxic effects of H_2_O_2_ upon the bacteria studied, we measured the survival rates of the *mazG*-null strains *versus* the wild-type strains of *Msm* and *Mtb* under the same H_2_O_2_ treatment conditions as that for mutation analysis. The survival rate of the *mazG*-null *Msm* decreased only slightly after 5 hours of H_2_O_2_ treatment compared to that of the wild type, while in the *mazG*-null *Mtb*, no significant effect was observed (**Figure 1C**). These data suggest that other than the change of mutation frequencies, H_2_O_2_ treatment in this study did not induce other major physiological change affecting the survival of the bacilli. Because the wild-type *mazG* gene complements all of the defective phenotypes of the *mazG*-null mutants (**Figure 1**), the antimutator role of mycobacterial MazG is genetically established.

### Mycobacterial MazG prevents CG to TA mutation

It has been shown that incorporation of different oxidized dNTPs into DNA preferentially induces a specific spectrum of mutation, *e*.*g*., 8-oxo-dGTP leads to AT to CG mutation [35], [36] while 5-OH-dCTP induces GC to AT mutation [9], [10], [22]. Therefore, we compared the mutation spectra between the *mazG*-null *Msm* and its parental strains to infer the probable substrate of mycobacterial MazG.

We sequenced the cluster I region of the *rpoB* gene [37] from randomly isolated rifampicin-resistant colonies. All of the sequences contained single nonsynonymous nucleotide variations. Of these, >99% were located within the cluster I region (the remaining mutations occurred outside of the cluster I region) and caused mutations in the well characterized rifampicin-resistance mutation hot spots (**Table S2 and S3**). Of the mutations detected, the frequency of CG to TA mutation exhibited a significant difference between the wild type and the *mazG*-null mutant (**Table 1**). Among the rifampicin-resistant mutants derived from the exponential-phase cells, the CG to TA mutation frequency in wild-type *Msm* increased from 2.2×10^−8^ in the untreated samples to 6.8×10^−8^ in the H_2_O_2_ treated cultures (∼3-fold increase), while in the *mazG*-null *Msm*, the frequency of this type of mutations increased significantly from 0.8×10^−8^ to 14.3×10^−8^ (∼18-fold increase). Meanwhile, the rifampicin-resistant mutants of the wild-type *Msm* exhibited a CG to TA mutation frequency in the stationary-phase cells similar to that in the exponential-phase cells (1.9×10^−8^ and 2.2×10^−8^, respectively), suggesting that the CG to TA mutation rate is likely constant during replication in the wild-type *Msm*. However, the CG to TA mutation in the *mazG*-null *Msm* increased 26-fold, from 0.8×10^−8^ in the exponential-phase cells to 20.5×10^−8^ in the stationary-phase cells (**Table 1**). These results clearly suggest that mycobacterial MazG is involved in safeguarding genomic DNA by preventing CG to TA mutation under adverse growth conditions.

**Table 1:** *mazG*-null *Msm* exhibited elevated CG to TA mutation under oxidative stress conditions and in the stationary phase of growth.

Growth phase	Strain (*n*)	Mutation frequency ×10^−8^ (*n*)							
		CG→TA	CG→AT	CG→GC	GC→CG	GC→TA	AT→GC	AT→CG	AT→TA
exponential	wt (30)	2.2 (17)	(0)	(0)	(0)	0.5 (4)	1.2 (9)	(0)	(0)
	wt+H_2_O_2_ (40)	6.8 (27)	(0)	(0)	(0)	1.2 (5)	0.8 (3)	1.2 (5)	(0)
	Δ*mazG* (43)	0.8 (18)	0.05 (1)	(0)	0.05 (1)	0.09 (2)	0.7 (14)	0.3 (7)	(0)
	Δ*mazG+*H_2_O_2_ (69)	14.3 (57)	(0)	(0)	(0)	(0)	2.0 (8)	1.0 (4)	(0)
stationary	wt (47)	1.9 (8)	(0)	3 (13)	(0)	5.1 (22)	0.2 (1)	(0)	0.7 (3)
	Δ*mazG* (45)	20.5 (34)	1.9 (3)	4.7 (8)	(0)	0.6 (1)	(0)	(0)	(0)

Spontaneous rifampicin-resistant colonies were collected from 3 independent experiments (see Methods). Cluster I region of *rpoB* were PCR-amplified using *pfu* DNA polymerase and sequenced bi-directionally. All of the sequenced colonies contain single non-synonymous mutations (see also **Table S1 and S2**). wt, wild-type *Msm*; Δ*mazG*, *mazG*-null *Msm*.

### 5-OH-dCTP is a preferred substrate for mycobacterial MazG

CG to TA transition, the most common base substitution occurring in aerobic organisms [38], [39], can be induced by incorporation of oxidatively damaged nucleotides into DNA, especially oxidized pyrimidine nucleotides [9], [10], [22]. We measured the MazG NTP-PPase activity towards 5-OH-dCTP, 5-CHO-dUTP and 2-OH-dATP, all of which are known to induce CG to TA mutation when incorporated into DNA [9], [22], [40].

**Figure 2:**
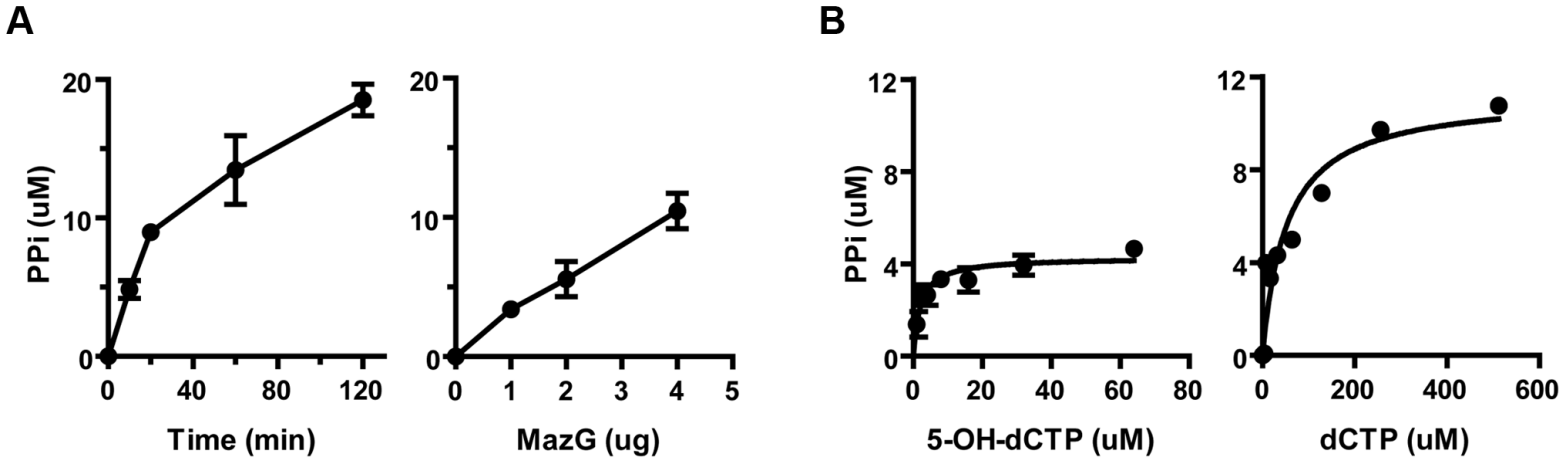
The NTP-PPase activity of mycobacterial MazG against 5-OH-dCTP. (**A**) Time- and enzyme concentration-dependent hydrolysis of 5-OH-dCTP. 5-OH-dCTP (200 µM) was incubated with 1 µg or varied amounts (from 1 µg to 4 µg) of heterogeneously expressed MazG purified to nearly SDS-PAGE homogeneity. The reaction was carried out at 37°C and terminated after 10 min or at the time points as indicated. PPi, pyrophosphate. Shown are mean ± S.E. of 3 repeats. (**B**) Michaelis-Menten curves of MazG with 5-OH-dCTP or dCTP as substrate. The hydrolytic product PPi is shown as µM/10 min. Data shown are mean ± S.E. of 3 independent experiments.

Each substrate was mixed with mycobacterial MazG proteins of different origin and was incubated at 37°C for 10 minutes. The hydrolyzed product, pyrophosphate, was quantified by an enzyme coupled colorimetric method [28]. It was found that all of these substrates were hydrolyzed into monophosphate derivatives and pyrophosphate in a time- and enzyme concentration-dependent manner (**Figure 2A**). Of the nucleotides examined, 5-OH-dCTP and 2-OH-dATP were the most preferred substrates for the *Mtb* MazG, with *K_m_* values of 1.9 and 2.4 µM, respectively, approximately 26 times lower than that of their canonical nucleotides (**Table 2** **and** **Figure 2B**). It appears that 5-CHO-dUTP is unlikely to be the natural substrate of *Mtb* MazG, shown by its extremely high *K_m_* value (∼500 µΜ, **Table 2**). The *Msm* MazG exhibited similar kinetic constants compared to its *Mtb* counterpart, except for 2-OH-dATP, which showed a *K_m_* of 311 µΜ (**Table S4**), much higher than that of the *Mtb* MazG (**Table 2**). Based on the kinetic constants and the same antimutator role of MazG in *Msm* and *Mtb*, we conclude that 5-OH-dCTP is the most likely natural substrate of mycobacterial MazG.

**Table 2:** Kinetic constants of *Mtb* MazG.

Substrate	*K_m_*	*k_cat_*	*k_cat_*/*K_m_* (×100)
	µM	min^−1^	min^−1^ µM^−1^
5-OH-dCTP	1.9±0.3	1.3	68
2-OH-dATP	2.4±0.7	1.9	79
5-CHO-dUTP	497±140	56.2	11
d-ATP	64±23	5.6	9
d-CTP	51±14	3.2	6

### 5-OH-dCTP is an *in vivo* substrate of mycobacterial MazG

To further characterize the natural substrate of mycobacterial MazG under cellular physiological conditions, we compared the *in vivo* mutagenic effects of these oxidized nucleotides in wild-type and *mazG*-null *Msm* strains using an established *in vivo* incorporation assay [22], [36].

**Figure 3:**
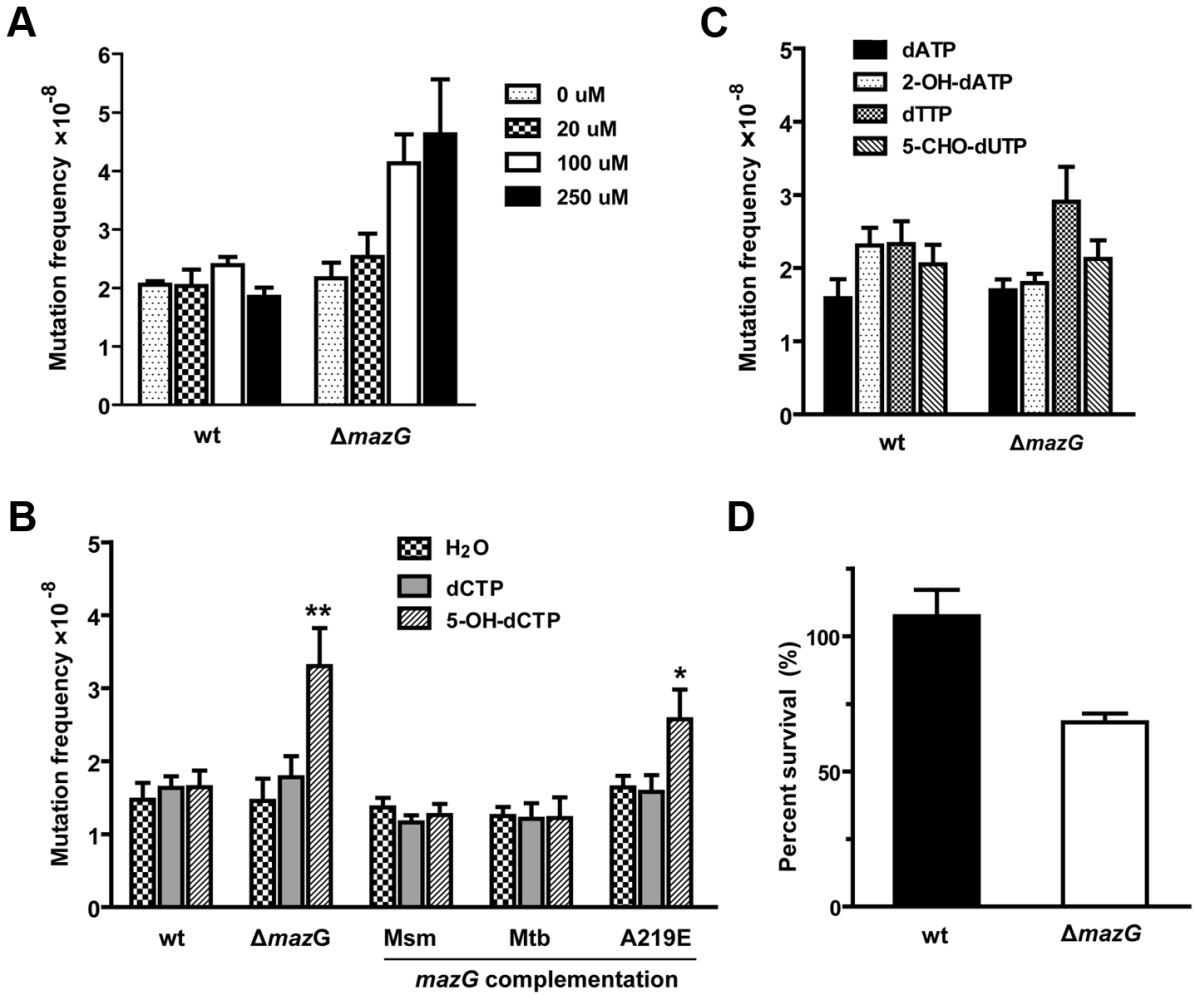
5-OH-dCTP is an *in vivo* substrate of mycobacterial MazG proved by chemical genetic analysis. The *Msm* competent cells were prepared as described in ***Materials and Methods***, the nucleotides were incorporated by transformation. (**A**) *mazG*-null *Msm* (Δ*mazG*) exhibited a dose-dependent 5-OH-dCTP induced mutagenesis. wt, wild-type *Msm*. The data shown are mean ± S.E. of four repeats. (**B**) The rifampicin-resistance mutation frequencies of wild-type *Msm*, the *mazG*-null mutant and the complemented mutant treated with 5-OH-dCTP and dCTP. Nucleotides were added at 100 µM final concentration. Mean ± S.E of 12 independent transformations. **p<0.01 *vs* wt, * *p*<0.05 *vs* wt. (**C**) The rifampicin-resistance mutation frequencies of wild-type and *mazG*-null *Msm* treated with 100 µM 2-OH-dATP, 5-CHO-dUTP and normal dNTPs. Mean ± S.E of 8 independent transformations. (**D**) *mazG*-null *Msm* is susceptible to killing by 5-OH-dCTP. Strains were treated with 100 µM 5-OH-dCTP for 5 hours at 37°C. Shown is percent survival compared to an untreated control (100%). The data shown are mean ± S.E. of four repeats.

Of the nucleotides tested, only 5-OH-dCTP exhibited a mutagenic effect upon the *mazG*-null *Msm* in a dose dependent manner (**Figure 3A–C**). When treated with 100 µM 5-OH-dCTP, the *mazG*-null mutant showed a ∼2 fold increase (P<0.01) in rifampicin-resistance mutation frequency compared to that of the wild-type *Msm* (**Figure 3B**). The increased mutation frequency of the *mazG*-null mutant can be restored to normal by complementation with a single copy of the wild-type *mazG* from either *Msm* or *Mtb*, indicating that MazG plays the same role in these two mycobacteria species (**Figure 3B**). Therefore, the antimutator role of mycobacterial MazG, particularly related to 5-OH-dCTP induced mutagenesis, is inferred. Furthermore, expression of the loss-of-function A219E MazG variant [28], [41] in *mazG*-null *Msm* failed to restore the mutator phenotype (**Figure 3B**), implying that the *in vivo* antimutator role of MazG requires NTP-PPase activity. We also found that the *mazG*-null *Msm* was susceptible to killing by 5-OH-dCTP treatment (**Figure 3D**), suggesting that mycobacterial MazG was involved in the defense against both the cytotoxic and the mutagenic effects of 5-OH-dCTP. Based on these biochemical and chemical genetic results, we conclude that 5-OH-dCTP is one of the *in vivo* substrates of mycobacterial MazG. However, we cannot exclude the possibility that other substrates may also exist.

### The *mazG*-null *Mtb* is hypersensitive to RNS

**Figure 4:**
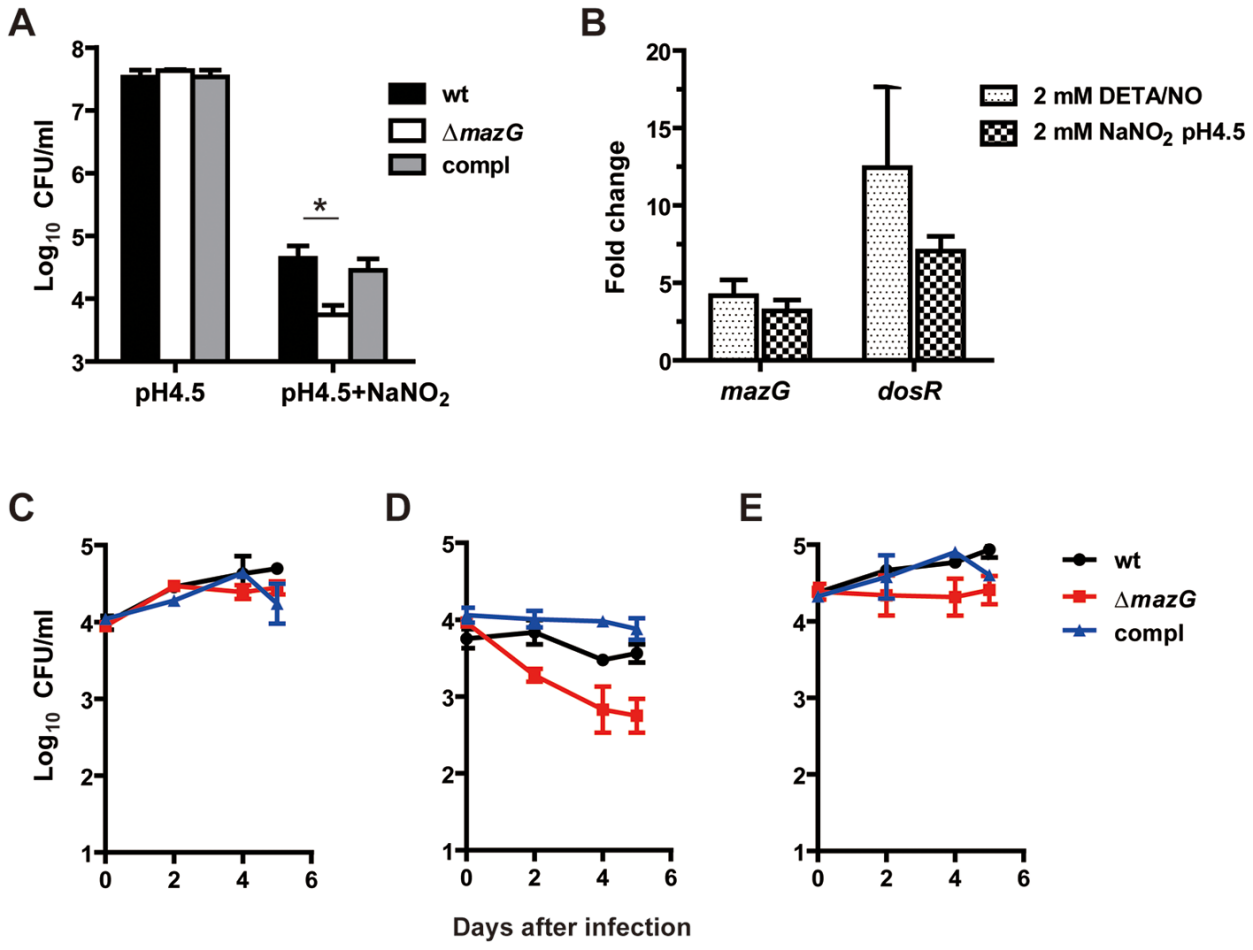
*Mtb* MazG is required for resistance to RNS shown *in vitro* and *ex vivo*. (**A**) *mazG*-null *Mtb* is susceptible to killing by acid nitrite. Exponential phase cultures (OD_600_∼0.5) were suspended in 7H9-OADC pH4.5 with or without 2.5 mM NaNO_2_ and treated for 20 hours. wt, wild-type *Mtb*; Δ*mazG*, *mazG*-null *Mtb*; compl, the complemented mutant. Data shown are mean ± S.E. of triplicates. * *p*<0.05 *vs* wt. (**B**) Quantitative real-time PCR analyses of *mazG* and *dosR* from *Mtb* treated with DETA/NO or acid nitrite. All data were normalized to the levels of *sigA*. The *dosR* gene was used as a positive control [70]. Results are expressed as the changes in expression levels compared to untreated samples. Mean ± S.E of three independent repeats. **C to E**, Survival of wild-type *Mtb*, the *mazG*-null mutant and the complemented mutant in resting (**C**), activated (**D**) or NMMA treated (**E**) cells of murine macrophage cell line RAW264.7. Shown is one representative result of two independent experiments.

During intracellular infection, *Mtb* is exposed to genetic assaults elicited by both ROS and RNS produced by host macrophages [5], [42]. We tested whether the MazG housecleaning function is involved in *Mtb* resistance to ROS and RNS. The *mazG*-null *Mtb* was found to be more susceptible to killing by acidified nitrite treatment *in vitro* than the wild-type *Mtb*, showing a 0.8-log_10_ lower CFU. The reduced survival ability of the *mazG* mutant can be fully restored by expression of a single copy of the wild-type *mazG* in the mutant (**Figure 4A**). Accordingly, transcription of the *mazG* gene in the wild-type *Mtb* was upregulated 3∼5 fold by the treatment of acid nitrite or DETA/NO (2,2-(hydroxynitrosohydrazino)-bisethanamine), which liberates nitric oxide (**Figure 4B**), indicating that *mazG* is involved in the genetic response to RNS. However, unlike the *mazG*-null *Msm* [28], the *mazG*-null *Mtb* was just as susceptible to H_2_O_2_ as the wild type (**Figure 1C**), a property shared by other *Mtb* mutants which are more sensitive to RNS *in vitro* [43], [44].

We further compared the intracellular survival ability between wild-type *Mtb* and the *mazG*-null mutant. No difference was observed between the growth of these two strains in resting macrophages (**Figure 4C**). However, when infected with activated macrophages, the titer of the *mazG*-null *Mtb* declined from 2 days post-infection and onward (**Figure 4D**), showing 1 log_10_ lower CFU than that of wild-type *Mtb* by 5 days post-infection. This suggests that MazG is required for *Mtb* resistance to intracellular RNS. Consistent with this finding, the attenuated survival of *mazG*-null *Mtb* in activated macrophages was partially rescued by addition of NMMA (N^G^-Methyl-L-arginine acetate salt), a specific inhibitor for macrophage inducible NO synthase (**Figure 4E**) [45]. Introduction of a wild-type *mazG* into the mutant strain restored the attenuated phenotype of the *mazG*-null *Mtb* (**Figure 4D–E**).

### MazG is required for *Mtb* survival *in vivo*

**Figure 5:**
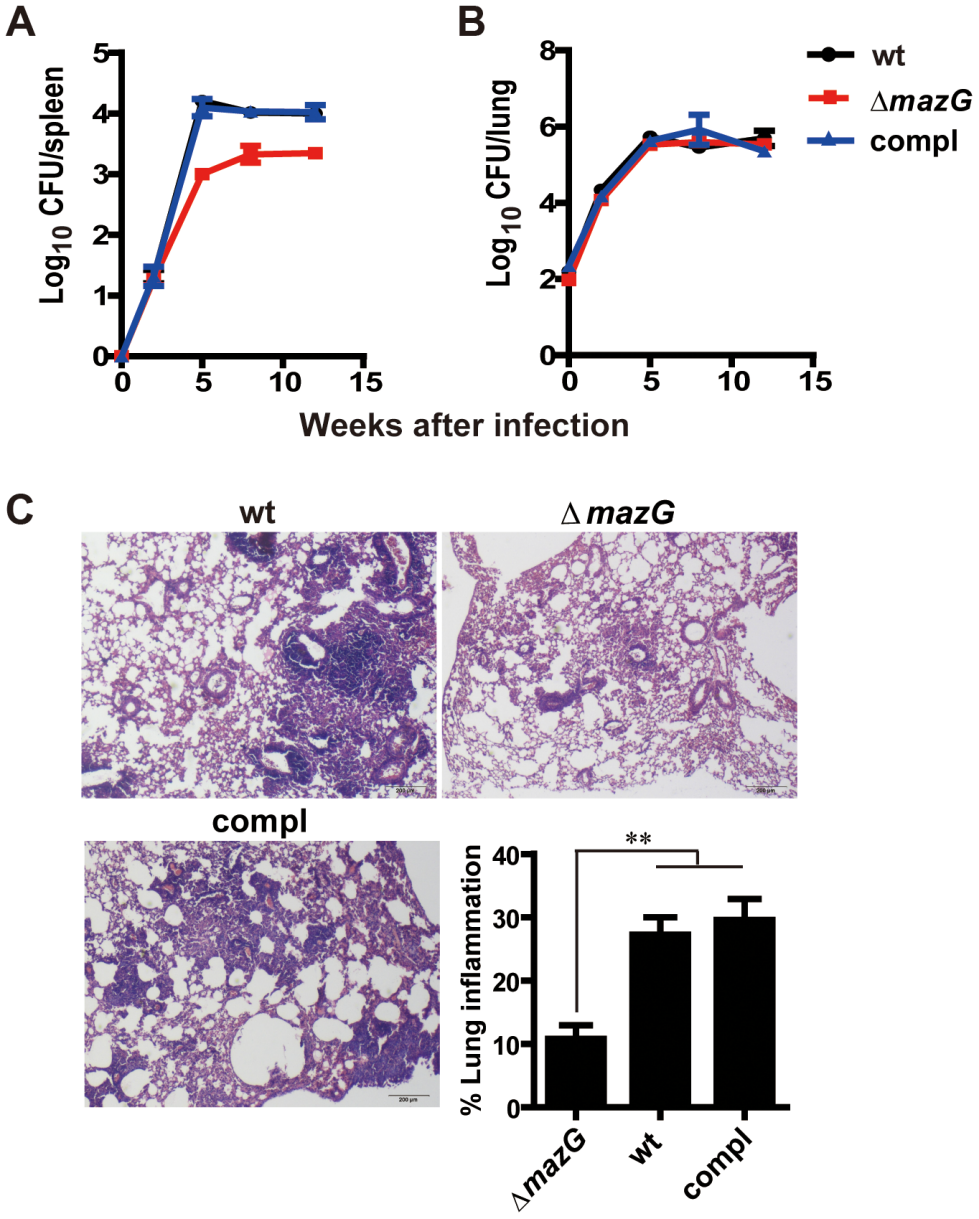
MazG is required for *Mtb* survival *in vivo* and the corresponding lung pathogenesis. **A to B**, Bacterial loads in spleen (**A**) and lung (**B**) of mice infected with wild-type *Mtb* (wt), the *mazG*-null mutant (Δ*mazG*) and the complemented mutant (compl). Data shown are mean ± S.E from 4 mice per group. (**C**) Lung sections taken from mice at 8-wk after infection and stained with hematoxylin and eosin. Inserted column shows mean ± S.E of lung inflammation of each group.

Our results demonstrate that *Mtb* MazG is required for maintenance of genetic stability and resistance to RNS both *in vitro* and *ex vivo*, indicating that MazG may function as a virulence factor during *Mtb* infection. To investigate whether *mazG* is involved in survival of *Mtb in vivo*, immune-competent mice were infected by a low-dose aerosol challenge with *Mtb* strains. Compared to wild-type *Mtb*, the *mazG*-null *Mtb* exhibited 1.1-log_10_ lower CFU in mice spleens by 4 weeks post-infection, and 0.7-log_10_ lower CFU by 8 and 12 weeks post-infection, indicating an attenuation at the stage of persistent infection (**Figure 5A**). No significant difference between the growth of wild-type and the *mazG*-null *Mtb* strains was observed in mice lung (**Figure 5B**). However, histological stained sections of the infected lung tissue (8 weeks after infection) showed that the *mazG*-null *Mtb* caused minimal pneumonitis, with a 3-fold reduction in lung inflammation compared to wild-type *Mtb* (**Figure 5C**). The attenuated phenotype of the *mazG*-null mutant can be fully restored by complementing the mutant with a single copy of the wild-type *mazG* (**Figure 5**). Taken together, these results suggest that the housecleaning role of MazG is required for *Mtb* survival and pathogenesis *in vivo*.

## Discussion

Growing evidence suggests that elimination of oxidized nucleotides from the cellular dNTP pool is an important safeguarding mechanism for maintenance of genetic stability [2], [3], [6], [7]. However, most of the knowledge about this housecleaning role has focused on oxidized purine nucleotides and the MutT-like NTP-PPase. Due to lack of knowledge of pyrimidine specific housecleaning NTP-PPase, the contribution of oxidized pyrimidine nucleotides to DNA mutagenesis and its related mechanism remains unclear. Here we characterized a NTP-PPase that specifically degrades 5-OH-dCTP *in vivo* and prevents CG to TA mutation. To our knowledge, MazG is the first oxidized pyrimidine-specific housecleaning enzyme to which an antimutator function can be assigned.

Our previous study suggested that 8-oxo-dGTP and dUTP are unlikely to be the natural substrates of mycobacterial MazG, as the *K_m_* values for these nucleotides are substantially high [28]. In this study, based on the observation that deletion of *mazG* leads to the increase of CG to TA mutation frequency, we considered 5-OH-dCTP, 5-CHO-dUTP and 2-OH-dATP as putative substrates of MazG, and all of these oxidized nucleotides are known to induce CG to TA mutations upon incorporated into DNA by polymerase [9], [22], [40]. Among the substrates tested, mycobacterial MazG showed high affinity towards 5-OH-dCTP (*K_m_* = 1.9 µM) and 2-OH-dATP (*K_m_* = 2.4 µΜ), values comparable to that of *E. coli* MutT towards its natural substrate 8-oxo-dGTP (*K_m_*∼0.5 µM) [8]. Through direct incorporation of these oxidized nucleotides into *Msm* cells, we observed a dose-dependent 5-OH-dCTP-specific mutagenic effect in *mazG*-null *Msm*, indicating 5-OH-dCTP is an *in vivo* substrate of mycobacterial MazG (**Figure 3**). Moreover, we also found that the mycobacterial MazG was involved in the defense against the cytotoxic effect of 5-OH-dCTP (**Figure 3D**). This cytotoxic effect is likely to be caused by lethal DNA strand breaks or replication block [11], [19] induced by incorporation of 5-OH-dCTP into DNA. Taken together, these data demonstrate that the mycobacterial MazG is a 5-OH-dCTP-specific housecleaning enzyme involved in preventing CG to TA mutation.

In *mazG*-null *Msm* with H_2_O_2_ treatment, we did not observe an increase of GC-TA mutations that should be induced by incorporation of 2-OH-dATP [46]. On the other hand, MazG does exhibit a high affinity to 2-OH-dATP, similar to its affinity to 5-OH-dCTP (**Table 2**). It is still unclear whether MazG is the only 2-OH-dATPase existing in mycobacteria. Therefore, whether 2-OH-dATP is a natural substrate of mycobacterial MazG remains unclear. It is worth noting that *Mtb* encodes four MutT proteins (MutT1 to 4) [47]. A recent study showed that the MutT1 carried out the physiological role of MutT (8-oxo-dGTPase) in *Mtb* [48], while the MutT2 did not function as an 8-oxo-dGTPase [49]. Therefore, to date, the natural substrate of MutT2-4 is still unidentified.

CG to TA transition can be induced by oxidative deamination of cytosines on DNA [50], misincorporation of oxidized pyrimidine nucleotides into DNA by DNA polymerase [9], [10], [22] and mismatch induced by keto-enol transitions of guanine [51]. Based on the biochemical and chemical genetic results described above, and the fact that MazG is unlikely to perform a DNA repair role, as this protein family does not contain any DNA binding/repair signatures [27], [28], we conclude that the increased CG to TA mutation in *mazG*-null *Msm* is mainly due to incorporation of oxidized nucleotide 5-OH-dCTP.

Deletion of *mazG* in mycobacteria did not lead to a mutator phenotype under the exponential phase of growth. However, lack of MazG activity in mycobacteria resulted in higher CG to TA mutation under both oxidative stress and the stationary growth phase, compared to that of the parental strains (**Table 1**). The likely mechanism underlying this stress-related mutagenesis is that under stress conditions, mycobacterial cells may accumulate 5-OH-dCTP and lacking MazG, more 5-OH-dCTP is misincorporated into DNA. Moreover, down-regulated DNA repair activity under these stress conditions [34], [52] may also contribute to the stress-related mutagenesis observed in the *mazG*-null strains. Nonetheless, although the related molecular mechanism of 5-OH-dCTP induced stress-related mutagenesis remains to be determined, as the host environment for *Mtb* parasitism is always adverse, 5-OH-dCTP induced mutagenesis may be hypothesized to play an important role in the microevolution process of the infected *Mtb* under stress conditions, *i.e.* emergence of drug resistant mutations during bacterial infection. In this connection, it is worth noting that CG to TA transition is a dominant mutation in *Mtb* isolated from either macaques with latent/reactivated infection or humans [53], [54].

It is still unclear why the lack of the 5-OH-dCTP sanitization function in *Mtb* results in hypersusceptibility to RNS (**Figure 4**). RNS is a group of radicals derived from nitric oxide (NO^•^) which are produced by macrophage as antimicrobial effector molecules [5]. An important antimicrobial action of RNS is inhibition of DNA replication and repair. It was found that NO^•^ can inhibit DNA synthesis by zinc mobilization from DNA-binding metalloproteins [55]. RNS can also inhibit ribonucleotide reductase [56], and thus, limit the availability of precursors for the synthesis and repair of DNA. Based on the fact that lack of MazG activity leads to increased incorporation of 5-OH-dCTP into DNA (**Table 1**), a possible explanation for the hypersusceptibility of *mazG*-null *Mtb* to RNS is that inhibition of DNA repair activities and lack of DNA precursors caused by RNS mediated enzyme inactivation result in higher levels of genetic instability (such as DNA strand breakage) in *mazG*-null *Mtb* than that in the wild-type *Mtb*.

During infection, *Mtb* is exposed to an oxidative environment of host macrophages rich in DNA-damaging ROS and RNS. Therefore, safeguarding of the genetic information is essential for mycobacterial survival, especially during the non-replicating dormancy stage, as slow or non-replicated genomic DNA and diminished DNA repair activities are likely lead to more genetic assaults than that during fast growing phase [52], [57]. Our results demonstrated that deletion of *mazG* leads to attenuated survival of *Mtb* in mice spleen during the persistent infection phase, suggesting that oxidative damage to nucleotides and the subsequent genetic assault is one of the bactericidal effects of the adaptive immune response (corresponding to the bacterial persistent infection stage). This is consistent with the data indicating that genes involved in removal of oxidized pyrimidines are essential for *Mtb* survival during primates' infection [58]. Although the difference between the lung and spleen microenvironments exposed to *Mtb* is unclear, it is conceivable that the immune responses and metabolic constraints are different between the two tissues. Interestingly, tissue specific attenuation have been demonstrated for several *Mtb* mutants, including the *dosR*, *fadD26*, *mptpB* and *narG* mutants [59]–[62].

Recent studies have proven that bactericidal antibiotics-induced ROS production within bacterial cells is a common mechanism for cell death [12], [63]–[65], predominantly elicited by incorporation of 8-oxo-dGTP into DNA [11]. Therefore, it is not surprising that 5-OH-dCTP and other oxidized nucleotides have a similar bactericidal effect, as shown in our results (**Figure 3D** **and** **Figure 5A**). These findings suggest that clinical treatment of tuberculosis with specific inhibitors of housecleaning enzymes might facilitate *Mtb* elimination, especially when combined with bactericidal antibiotics which are known to induce oxidative stress.

## Materials and Methods

### Ethics statement

Six-to-eight week old female C57BL/6 mice were purchased from the Shanghai SLAC Laboratory Animal Company. The mice were housed and cared for in a specific pathogen-free (SPF) biosafety level 3 facility at Shanghai Public Health Clinical Center. Mice were provided food and water *ad libitum* as well as appropriate monitoring and clinical care. Animal experiments were carried out in strict accordance with the regulations in the Guidance Suggestions for the Care and Use of Laboratory Animals issued by the Ministry of Science and Technology of the People's Republic of China. The protocol was approved by the Chinese Science Academy Committee on Care and Use of Laboratory Animals and the Laboratory Animal Ethical Board of Shanghai Public Health Clinical Center (Permit Number: 2012A002).

### Bacterial strains and culture conditions

Bacterial strains used in this study are list in **Table S1**. Bacterial culturing was performed as described [66]. *Msm* strains were grown at 37°C in 7H9 broth (BD Difco), or on Luria-Bertani agar supplemented with 0.5% glycerol (LBG agar). *Mtb* strains were grown at 37°C in 7H9 broth supplemented with 10% OADC (7H9-OADC), or on 7H11 plates supplemented with 10% OADC (7H11-OADC). When required, the following antibiotics were used at the specified concentrations: kanamycin (15 µg/ml), hygromycin B (150 µg/ml for *Msm* and 50 µg/ml for Mtb) and rifampicin (250 µg/ml for *Msm* and 10 µg/ml for *Mtb*). For treatment with acid NO, *Mtb* stains grown to OD_600_∼0.5 were pelleted and re-suspended in 7H9-OADC pH 4.5 (adjusted by 1 M citrate) with or without 2.5 mM NaNO_2_ [45]. After 20 h treatment, bacteria were plated on 7H11-OADC, CFUs were counted after 3∼4 weeks culture at 37°C

### Generation of *mazG* mutants and complemented strains

The *mazG*-null mutant was generated by the phage transduction method [66]. *mazG*-null *Msm* and the complemented strains were generated as described [28]. To construct a transducing phage for *Mtb mazG* knockout, the left homologue arm was PCR amplified using primers KOP1 and KOP2 (primers used in this study are listed in **Table S5**). The right homologue arm was PCR amplified using primers KOP3 and KOP4. The PCR products were ligated into the *Afl*II/*Xba*I and *Hin*dIII/*Xho*I sites of pYUB854. The recombinant transducing phage was used to construct the *mazG*-null *Mtb* as described [28]. The *mazG*-null mutant was verified by southern blot and PCR (**Figure S2**). The probe for Southern blot was PCR amplified using primers SB1and SB2. A dUTP-biotin labeled probe (Fermentas) was used for Southern blot analysis of the *Pst*I*/Kpn*I digested chromosomal DNA on the Hybond-N^+^ nylon membrane (GE Amersham), according to the standard method [67]. Primers used for genotyping PCR were P1, P2 and P3. The complementation plasmid for *mazG*-null *Mtb* was generated by ligating the PCR product amplified using primers C1 and C2 into the *Bam*HI and *Hin*dIII sites of pMV306. Expression of *Mtb mazG* was controlled by its own promoter (1142671–1143646).

### Determination of rifampicin-resistance mutation frequency

Single colonies of various *Msm* strains from the 7H11 agar plate were inoculated in 5 ml media and cultured at 37°C for 48 h (2 weeks for *Mtb* strains). For determination of rifampicin-resistance mutation frequency, the cultures were inoculated with 1% of primary culture in 20 ml 7H9 media (in a 100-ml flask) without antibiotics and grown at 37°C with rolling (150 rpm) to exponential phase (OD_600_∼0.5). Then 10 ml of the cultures were treated with 10 mM H_2_O_2_, and another 10 ml cultures were untreated. After incubation at 37°C, 150 rpm, for 5 h, CFU per ml was determined by plating; the cell pellet from 3 ml culture was plated on LBG agar (3 plates of each sample) containing 250 µg/ml rifampicin (Sigma-Aldrich). The CFU and rifampicin-resistant colonies were counted after culturing at 37°C for 4 days (28 days for *Mtb* strains). The rifampicin-resistance mutant frequency was calculated by dividing the number of rifampicin-resistant colonies on each plate by the counts of the total viable cells plated. Rifampicin-resistance mutation frequencies of *Mtb* strains were determined by the same method, except that the oxidative stress was elicited by resuspending the exponential-phase cell pellet in 7H9 media containing 10 mM H_2_O_2_, followed with incubating at 37°C for 24 h. *Mtb* strains were plated on 7H11-OADC with or without 10 µg/ml rifampicin. For determination of rifampicin-resistance mutation frequency of the stationary phase cultures, cells were cultured in liquid media for 5 days (for *Msm*) or 28 days (for *Mtb*) and plated as described above. Three independent experiments were performed with totaling 15 cultures of each *Msm* strains and 6 of *Mtb*.

### Analysis of mutation spectra

Rifampicin-resistant colonies were collected from three independent experiments. The isolated colonies were grown in 1 ml 7H9 at 37°C for 1 week. Cells were pelleted and suspended in 50 µl TE buffer (10 mM Tris–HCl, pH 8.0, and 1 mM EDTA) and incubated at 95°C for 10 min to extract the genome DNA [37]. The lysate was centrifuged at 12000 g for 5 min. The supernatant was used as template to amplify (using *pfu* DNA polymerase) the fragment containing the cluster I region of *rpoB* using primers Rpo1 and Rpo2. All PCR products were sequenced by bi-directionally. Mutation spectra of the sequenced region were analyzed by BioEdit software.

### NTP-PPase assay

Protein expression and purification was performed as described [28]. Protein was purified to nearly SDS-PAGE homogeneity. Protein concentration was determined by the bicinchoninic acid (BCA) method [68]. The oxidized nucleotides used as substrates for MazG were purchased from TriLink Biotechnologies Inc. (5-OH-dCTP) or Hongene Biotechnologies Inc. (2-OH-dATP and 5-CHO-dUTP). The NTP-PPase activity of MazG was assayed as described [28]. The NTP-PPase assay was carried out in 20 µl reaction buffer (20 mM Tris-HCl, pH 7.5, 5 mM MgSO_4_, 100 mM NaCl) containing 1 µg mycobacterial MazG and substrate nucleoside triphosphates at 37°C for 10∼20 min. The reaction was stopped by heating at 65°C for 5 min, and 10-20 µl products were applied for pyrophosphate assay (Molecular Probes) according to the manufacturer's instructions. Reactions with heat inactivated (95°C for 20 min) MazG protein were set up as a background controls. GraphPad Prism 5.0 (GraphPad Software, Inc.) was used for enzyme kinetic constants analysis.

### Incorporation of nucleotides into *Msm* competent cells

The *Msm* competent cells were prepared from 400-ml cultures (OD_600_ = 0.8∼1.0) as previously described [69]. Incorporation of nucleotides into *Msm* competent cells was performed as described [22], [36]. Briefly, Nucleotide solution (100 µM final concentration) was added to 150 µl competent cells suspension and the mixture was placed on ice for 10 min. After heat shock treatment (42°C for 90 sec and then on ice for 30 min), 2 ml 7H9 was added and the cells were incubated at 37°C with rolling (150 rpm) for 5 h. After treatment, 2 ml of culture was centrifuged at 4000 g for 5 min and plated on LBG agar containing 250 µg/ml rifampicin. The remaining culture was diluted and plated onto LBG agar for CFU determination. Rifampicin-resistance mutation frequencies were calculated as described above.

### RNA extraction and quantitative real-time PCR

Wild-type *Mtb* (OD_600_∼0.5) was treated with acid NO or 2.5 mM DETA/NO for 1 h. Total RNA was extracted with TRIzol-Reagent (Invitrogen) and further purified with RiboPure-Bacteria kit (Ambion). Briefly, cell pellet was resuspended in 1 ml TRIzol reagent, mixed with 400 µl 0.1 mm Zirconia Beads (BioSpec Products) and lysed in a mini-beadbeater (Biospec) for three cycles (40 s at maximal speed) with cooling on ice for 1 min between pulses. RNA was extracted according to the protocol of TRIzol-Reagent. The extracted RNA was further purified using the RiboPure-Bacteria kit followed by DNase I treatment to eliminate DNA contamination. cDNA was synthesized using the SuperScript III First Strand kit (Invitrogen) with random hexamer primer. Target gene transcript levels were measured by real-time PCR using SYBR® Premix Ex Taq GC (TaKaRa) on Mastercycler ep realplex thermal cyclers: 95°C 60 sec, 40 cycles of 95°C 5 sec, 62°C 8 sec and 72°C 20 sec, followed by melting curve analysis. Data were normalized to *sigA* and expressed as fold change compared to the untreated samples. PCR primers for *sigA*, *mazG*, *dosR*, *recA* and *dnaE2* are listed in **Table S5**.

### Macrophage infection

The murine macrophage cell line RAW264.7 was grown in DMEM medium (GIBCO) supplemented with 10% fetal calf serum (FCS) and incubated at 37°C with 5% CO_2_. For *Mtb* infection, cells were plated at a density of 2.0×10^5^ cells per well in 24-well plates without antibiotics and activated with 200 U/ml murine IFN-γ (Peprotech) for 16 h [45]. Cells were primed with 1 µg/ml lipopolysaccharides (LPS, Sigma) for 1 h and then infected at a multiplicity of infection (MOI) of 2∶1 (bacteria∶cells). After 4 h incubation at 37°C with 5% CO_2_, cells were washed three times with DMEM to remove extracellular bacteria and cultured with complete DMEM medium. To inhibit macrophage NO production during the infection cause, NMMA (Sigma) was added to the culture medium at a final concentration of 400 µM. At indicated time points, bacteria were released with PBS solution containing 0.05% Tween-80 and 0.025% SDS, and plated onto 7H11-OADC plates. CFUs were counted after 3∼4 weeks culture at 37°C.

### Mice infection

Mice were infected with wild-type *Mtb*, *mazG*-null mutant or complemented strain at an inhaled aerosol dose of 100-200 CFU per lung by an inhalation exposure system (Glas-Col, Terre Haute, IN). At indicated time points, mice were sacrificed and lung and spleen homogenates (four mice per group) were plated onto 7H11-OADC followed by incubation at 37°C for 4 weeks.

### Histopathology

Lung sections stained with hematoxylin and eosin were photographed using a Nikon Optiphot 2 microscope fitted with a camera which was connected to a computer. The Image Pro Plus program (Media Cybernetics) was utilized to objectively assess the level of inflammation present in each image. To quantify the percent area inflamed, we determined the mean percent inflamed area from three to five lung sections of each mouse.

### Statistical analysis

Statistical significance was determined with the unpaired two-tailed Student's t test at P<0.05 level of significance using GraphPad Prism 5.0 software.

## Supporting Information

Figure S1 ***mazG*** **-null** ***Msm*** **exhibited higher level of SOS response under oxidative stress.** Expression level of *recA* and *dnaE2* from exponential phase bacteria and oxidative stressed samples (treated with 10 mM H_2_O_2_ for 1 h) were measured by quantitative real-time PCR and normalized to *sigA*. Shown are fold change compared to the untreated samples. wt, wild-type *Msm*; Δ*mazG*, *mazG*-null *Msm*. Mean± S.E. of three independent repeats.
(TIF)

Figure S2 **Characterization of** ***mazG*** **-null** ***Mtb*** **.** (A) Schematic diagrams of wild-type (wt) and the *mazG*-null (Δ*mazG*) loci. The primers used for PCR are shown as arrows. (B) Southern blot analysis of wt *Mtb* and the Δ*mazG* mutant. A dUTP-biotin labeled fragment was used to probe PstI/KpnI-digested chromosomal DNA separated by 0.8% agarose gel. Sizes of DNA bands are as indicated. (C) Analysis of PCR products from wt *Mtb* and the Δ*mazG* mutant. C1 and C2 are two hygromycin-resistant colonies.
(TIF)

Table S1 **Bacteria strains used in this study.**
(PDF)

Table S2 **Codon mutations determined in exponential phase** ***Msm*** **-derived rifampicin-resistant mutant.** Codon 427,429, 432 and 442 are rifampicin-resistant hot spots of *rpoB*.
(PDF)

Table S3 **Codon mutations determined in stationary phase (5-day)** ***Msm*** **-derived rifampicin-resistant mutant.** All listed codons are rifampicin-resistant hot spots of *rpoB*.
(PDF)

Table S4 **Kinetic constants of** ***Msm*** **MazG.**
(PDF)

Table S5 **Primers used in this study.**
(PDF)
