# Supplementary figures and images for: Mycobacterial MazG Safeguards Genetic Stability via Housecleaning of 5-OH-dCTP
Source: PLoS Pathog. 2013 Dec 5;9(12):e1003814. doi: 10.1371/journal.ppat.1003814 (PMC3855555; doi:10.1371/journal.ppat.1003814)

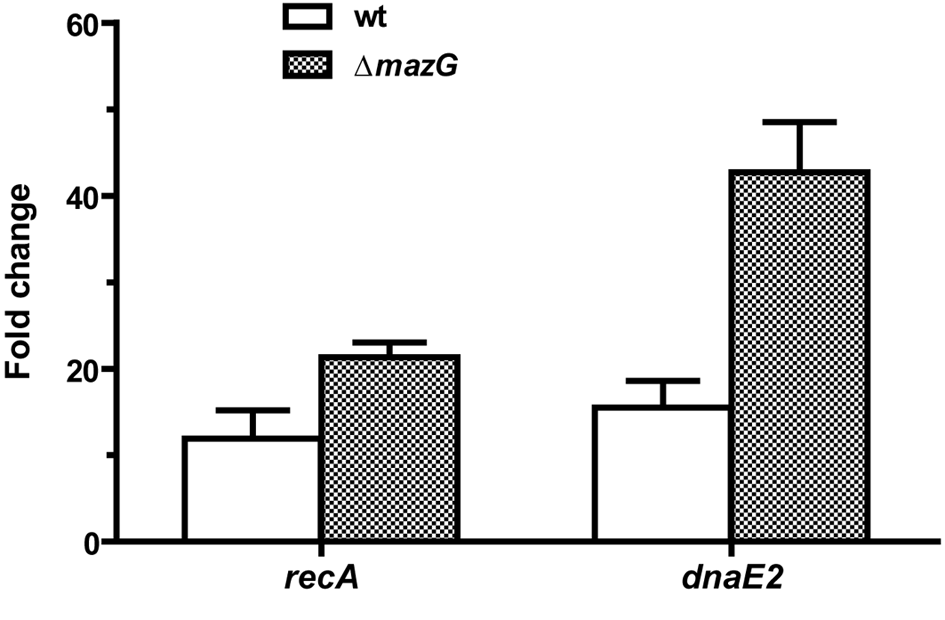

Supplement: Figure S1 — mazG -null Msm exhibited higher level of SOS response under oxidative stress. Expression level of recA and dnaE2 from exponential phase bacteria and oxidative stressed samples (treated with 10 mM H2O2 for 1 h) were measured by quantitative real-time PCR and normalized to sigA. Shown are fold change compared to the untreated samples. wt, wild-type Msm; ΔmazG, mazG-null Msm. Mean± S.E. of three independent repeats. (TIF) [file ppat.1003814.s001.tif]

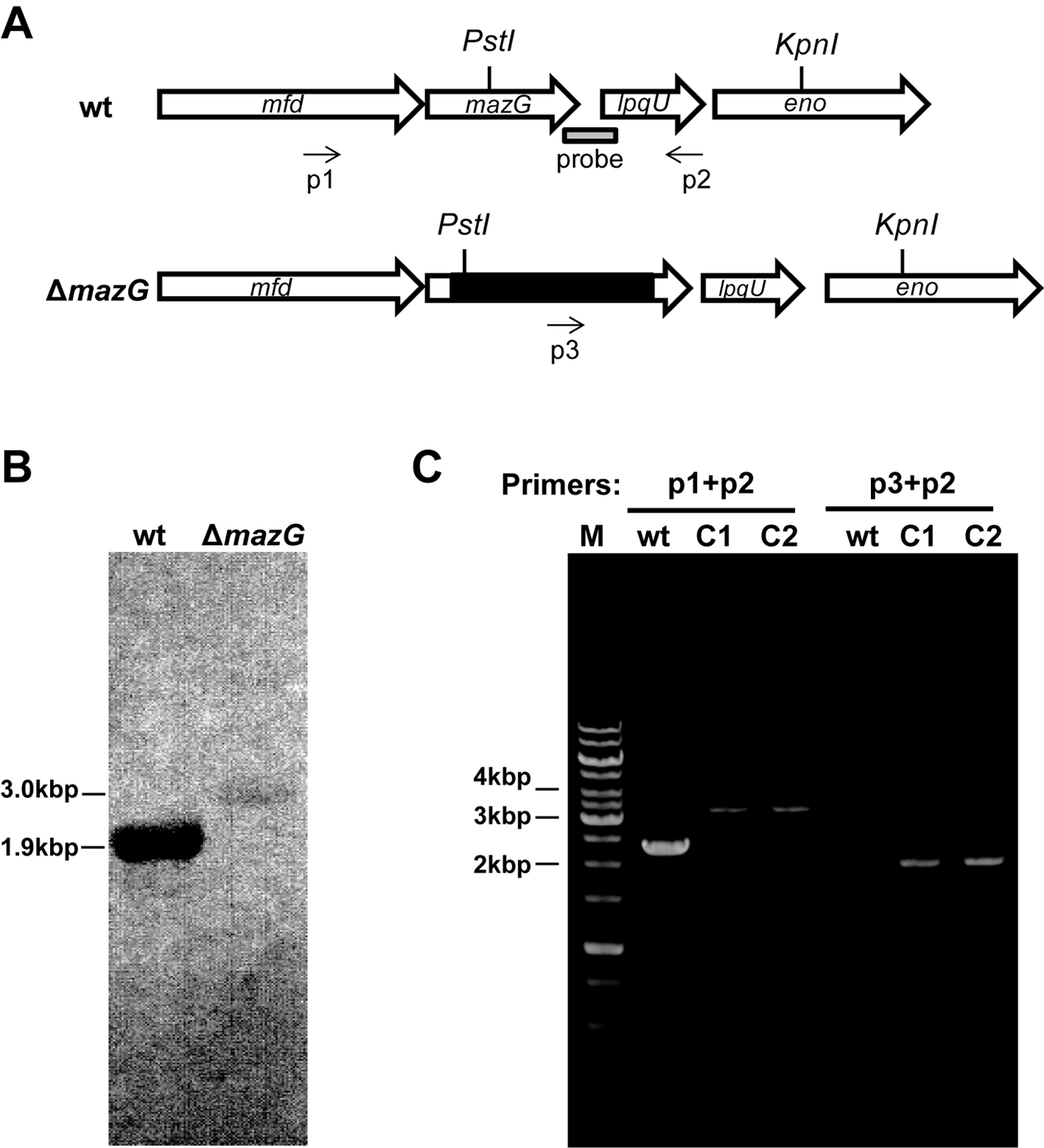

Supplement: Figure S2 — Characterization of mazG -null Mtb . (A) Schematic diagrams of wild-type (wt) and the mazG-null (ΔmazG) loci. The primers used for PCR are shown as arrows. (B) Southern blot analysis of wt Mtb and the ΔmazG mutant. A dUTP-biotin labeled fragment was used to probe PstI/KpnI-digested chromosomal DNA separated by 0.8% agarose gel. Sizes of DNA bands are as indicated. (C) Analysis of PCR products from wt Mtb and the ΔmazG mutant. C1 and C2 are two hygromycin-resistant colonies. (TIF) [file ppat.1003814.s002.tif]
