# Supplementary material for: Mycobacterial MazG Safeguards Genetic Stability via Housecleaning of 5-OH-dCTP
Source: PLoS Pathog. 2013 Dec 5;9(12):e1003814. doi: 10.1371/journal.ppat.1003814 (PMC3855555; doi:10.1371/journal.ppat.1003814)
Supplement: Table S1 — Bacteria strains used in this study. (PDF) [file ppat.1003814.s003.pdf]

Table S1. Bacteria strains used in this study

**Strain (Lab ID)**	**Relevant genetic background information and construction details**	**Source or Reference**
Mtb (4-187)	*M. tuberculosis* H37Rv, with wild-type *mazG*	ATCC
mazG-null *Mtb* (4-188)	*Mtb* wherein the *mazG* gene is replaced with *hyg* cassette (H37Rv Δ*mazG*::*hyg*)	This work
mazG-null *Mtb* /pMV306mt (4-189)	*mazG*-null *Mtb* with an integrative plasmid pMV306 expressing *Mtb mazG*	This work
Msm (4-190)	*M. smegmatis* mc^2155, with wild-type *mazG*	ATCC
mazG-null *Msm* (4-191)	*Msm* wherein the *mazG* gene is replaced with *hyg* cassette (mc^2155 Δ*mazG*::*hyg*)	Ref. 28
mazG-null *Msm*/pMV306ms (4-192)	*mazG*-null *Msm* with an integrative plasmid pMV306 expressing *Msm mazG*	Ref. 28
mazG-null *Msm*/pMV306mt (4-193)	*mazG*-null *Msm* with an integrative plasmid pMV306 expressing *Mtb mazG*	Ref. 28
mazG-null *Msm*/pMV306A219E (4-194)	*mazG*-null *Msm* with an integrative plasmid pMV306 expressing the A219E variant of *Mtb mazG*	Ref. 28
