# Supplementary material for: Mycobacterial MazG Safeguards Genetic Stability via Housecleaning of 5-OH-dCTP
Source: PLoS Pathog. 2013 Dec 5;9(12):e1003814. doi: 10.1371/journal.ppat.1003814 (PMC3855555; doi:10.1371/journal.ppat.1003814)
Supplement: Table S2 — Codon mutations determined in exponential phase Msm -derived rifampicin-resistant mutant. Codon 427,429, 432 and 442 are rifampicin-resistant hot spots of rpoB. (PDF) [file ppat.1003814.s004.pdf]

Table S2. Codon mutations determined in exponential phase *Msm*-derived rifampicin-resistant mutant. Codon 427, 429, 432 and 442 are rifampicin-resistant hot spots of *rpoB*.

Sample	Number of codon mutation (%)									
	[442]CAC- TAC	[442]CAC-CGC	[442]CAC-CCC	[429]CAG-AAG	[429]CAG-TGG	[460]GGC-CGC	[465]CCG-TCG	[427]CAA-CAC	[466]CAT-CGT	[432]GAC-TAC
wt	17 (57)	9 (30)	0	0	0	0	0	0	0	4 (13)
wt+H_2O_2	27 (68)	3 (8)	5 (12)	0	0	0	0	0	0	5 (12)
Δ*mazG*	17 (40)	14 (33)	6 (14)	1 (2)	0	1 (2)	1 (2)	1 (2)	0	2 (5)
Δ*mazG*+H_2O_2	56 (82)	6 (9)	4 (6)	0	1 (2)	0	0	0	1 (2)	0
