# Supplementary material for: Mycobacterial MazG Safeguards Genetic Stability via Housecleaning of 5-OH-dCTP
Source: PLoS Pathog. 2013 Dec 5;9(12):e1003814. doi: 10.1371/journal.ppat.1003814 (PMC3855555; doi:10.1371/journal.ppat.1003814)
Supplement: Table S3 — Codon mutations determined in stationary phase (5-day) Msm -derived rifampicin-resistant mutant. All listed codons are rifampicin-resistant hot spots of rpoB. (PDF) [file ppat.1003814.s005.pdf]

Table S3. Codon mutations determined in stationary phase (5-day) *Msm*-derived rifampicin-resistant mutant. All listed codons are rifampicin-resistant hot spots of *rpoB*.

Sample	Number of codon mutation (%)									
	^{432}**G**AC-**T**AC	^{432}G**A**C-G**G**C	^{447}T**C**G-T**G**G	^{447}T**C**G-T**T**G	^{438}T**C**G-T**G**G	^{438}T**C**G-T**T**G	^{442}C**A**C-C**T**C	^{442}**C**AC-**G**AC	^{442}**C**AC-**T**AC	^{442}**C**AC-**A**AC
wt	22 (46)	1 (2)	11 (23)	6 (13)	0	1 (2)	3 (6)	2 (4)	1 (2)	0
Δ*mazG*	1 (2)	0	6 (13)	32 (70)	1(2)	0	0	1 (2)	2 (4)	3 (7)
