# Supplementary material for: Mycobacterial MazG Safeguards Genetic Stability via Housecleaning of 5-OH-dCTP
Source: PLoS Pathog. 2013 Dec 5;9(12):e1003814. doi: 10.1371/journal.ppat.1003814 (PMC3855555; doi:10.1371/journal.ppat.1003814)
Supplement: Table S4 — Kinetic constants of Msm MazG. (PDF) [file ppat.1003814.s006.pdf]

Table S4. Kinetic constants of *Msm* MazG

**Substrate**	$\boldsymbol{K_m}$	$\boldsymbol{k_{cat}}$	$\boldsymbol{k_{cat}/K_m}$ (×100)
	μM	min^{-1}	min^{-1} $μM^{-1}$
5-OH-dCTP	5.6 ± 2.2	0.8	14
5-CHO-dUTP	434 ± 82	60	14
2-OH-dATP	311 ± 142	8.2	3
