# Supplementary material for: Mycobacterial MazG Safeguards Genetic Stability via Housecleaning of 5-OH-dCTP
Source: PLoS Pathog. 2013 Dec 5;9(12):e1003814. doi: 10.1371/journal.ppat.1003814 (PMC3855555; doi:10.1371/journal.ppat.1003814)
Supplement: Table S5 — Primers used in this study. (PDF) [file ppat.1003814.s007.pdf]

Table S5. Primers used in this study

Primer name	Sequences
KOP1	CTCTCCTTAAGAA GAACCCAAGGATGTGCG
KOP2	CTCTCTCTAGATCGCTTCAACAGGCACCAG
KOP3	CTCTCAAGCTTGCCAACTGCTG TCAACGATT
KOP4	CTCTCCTCGAGCAGGTTTCCGAAATGAACTG
SB1	GCGACGGGGTAGTCAACAA
SB2	CAGGTTTCCGAAATGAACTG
P1	CGCGCATAGATAACGGA GTTG
P2	GAGACGTACCGGGACGCGTAC
P3	TTCGGATGATTCCTACGCG
C1	AACCGGATCCGATGATTGTCGTCCTGGTCGAC
C2	AACCT AAGCTTATCGCATGCCTTTACGTTTC
Rpo1	CGACCACTTCGGCAACCG
Rpo2	CGATCAGACCGATGTTGG
SigA1	CTCGACGCTGAACCAGAC
SigA2	GAGGTCTTCGTGGTCTTCGT
MazG1	GATTTCGCTCGAAGATCAAC
MazG2	TTGGGCACGCTGAATA AC
DosR1	AGCTTGACGTCGTAGGTGAG
DosR2	AGGACGTGAGGAT CAGACAG
RecA1	GAGATCGAGGGCGAGATG
RecA2	GGCGTAGAACTTCAGTGCCT
DnaE2-1	CGAACGTCACGGCATCAC
DnaE2-2	CAGCGAGGCGAAGCTCAG
